# Inhibition effect of choline and parecoxib sodium on chronic constriction nerve injury-induced neuropathic pain in rats

**DOI:** 10.1186/s12871-022-01913-0

**Published:** 2023-01-13

**Authors:** Na Zhang, Yang Li, Zeguo Feng

**Affiliations:** 1 Anesthesiology Department, Civil Aviation General Hospital, Beijing, 100123 People’s Republic of China; 2 Chinese PLA Medical School, Beijing, 100853 China; 3 Department of Pain Medicine, First Medical Center, Chinese PLA General Hospital, No.28 Fuxing Road, Haidian District, Beijing, 100853 People’s Republic of China

**Keywords:** Neuropathic pain, Choline, Parecoxib sodium, HMGB1, NF-κB

## Abstract

**Purpose:**

The simultaneous use of drugs with different mechanisms of analgesic action is a strategy for achieving effective pain control while minimizing dose-related side effects. Choline was described to potentiate the analgesic action of parecoxib sodium at small doses in several inflammatory pain models. However, these findings are still very limited, and more associated data are required to confirm the effectiveness of the combined choline and parecoxib sodium therapy against inflammatory pain.

**Methods:**

Adult rats were randomly divided into 9 groups (*N* = 6/group). The sham surgery group received an intraperitoneal (i.p.) injection of saline. Rats with chronic constriction injury (CCI) of the sciatic nerve received saline, choline (cho, 6, 12 and 24 mg/kg), parecoxib sodium (pare, 3, 6, and 12 mg/kg), or a combination of choline 6 mg/kg and parecoxib sodium 3 mg/kg. Mechanical and heat pain thresholds were measured at 30 min after drug treatment at Days 3, 5, 7, 10, and 14 after CCI. Another 30 rats were divided into 5 groups (*N* = 6/group): the sham, CCI + saline, CCI + cho-6 mg/kg, CCI + pare-3 mg/kg, and CCI + cho-6 mg/kg + pare-3 mg/kg groups. After repeated drug treatment for 7 days, five rats were randomly selected from each group, and the lumbar dorsal root ganglia (DRGs) (L4–6) were harvested for western blot analysis.

**Results:**

Choline significantly attenuated mechanical and heat hypersensitivity in CCI rats at 12 and 24 mg/kg doses (*P* < 0.05) but was not effective at the 6 mg/kg dose. Parecoxib sodium exerted significant pain inhibitory effects at the 6 and 12 mg/kg doses (*P* < 0.05) but not at the 3 mg/kg dose. Combining a low dose of choline (6 mg/kg) and parecoxib sodium (3 mg/kg) produced significant pain inhibition in CCI rats and reduced the expression of high mobility group protein 1 (HMGB1) and nuclear factor-kappa Bp65 (NF-κBp65) in L4–6 DRGs.

**Conclusion:**

1. In a rat model of chronic neuropathic pain (CCI), at a certain dose, choline or parecoxib sodium can alleviate mechanical pain and thermal hyperalgesia caused by CCI. 2. The combination of choline and parecoxib sodium in nonanalgesic doses can effectively relieve neuropathic pain, and its mechanism may be related to the inhibition of the high mobility group protein 1 (HMGB1)/Toll-like receptor 4 (TLR4)/nuclear factor kappa-B (NF-κB) pathway.

**Supplementary Information:**

The online version contains supplementary material available at 10.1186/s12871-022-01913-0.

## Introduction

According to statistics, 1/3 of the global population is exposed to chronic, persistent or recurrent periodic pain. Pain not only reduces the function of various organs of the body but also has a great impact on the psychology of patients and reduces their quality of life. Hence, rational uses of drugs to prevent and control pain diseases are of great significance. Postoperative pain is the acute pain most often considered by clinical anesthesiologists. The use of single analgesic drugs or methods cannot achieve satisfactory results, and the use of combined drugs and multimode analgesia after surgery has gradually been recognized [1].

Neuropathic pain is a chronic disease caused by injury to the nervous system or functional disorders [2]. After nervous system injury, the release of inflammatory factors and mediators induces inflammatory responses. These changes may increase the excitability of nociceptive sensory neurons causing peripheral and central sensitization, which are involved in neural plasticity and contribute to the occurrence and maintenance of neuropathic pain [3].

Nonsteroidal anti-inflammatory drugs (NSAIDs) produce anti-inflammatory, antipyretic and analgesic effects by inhibiting cyclooxygenase (COX) activity and blocking the conversion of arachidonic acid into prostaglandin and thromboxane. As a specific COX-2 inhibitor, parecoxib sodium is an amino acid compound of the valdecoxib precursor, which is rapidly transferred to valdecoxib after intravenous injection. As a highly selective COX-2 inhibitor, valdecoxib has the smallest semi-inhibitory concentration. Multidisciplinary research has shown that parecoxib sodium is characterized by effective analgesia and low rates of side effects [4]. Hence, parecoxib sodium is an ideal clinical NSAID analgesic and has been widely used for postoperative analgesia. However, parecoxib sodium exhibits some side effects, including gastrointestinal system adverse reactions.

The nicotinic acetylcholine receptor (nAChR) mediates the excitation of neuromuscular junctions; participates in memory, cognition, and addiction; and regulates differentiation, proliferation and inflammatory responses. α7-Nicotinic acetylcholine receptor (α7nAChR) is necessary for the anti-inflammatory effect of acetylcholine [5]. The cholinergic anti-inflammatory pathway (CAP) is a new inflammatory regulatory mechanism. The CAP is an anti-inflammatory signal that binds to α7nAChR expressed on immune cells such as macrophages through released acetylcholine to inhibit the release of proinflammatory cytokines, thus playing an anti-inflammatory and analgesic role [6]. As a specific α7nAChR agonist, choline can inhibit the release of various proinflammatory cytokines by triggering the inhibition of α7nAChR, thus playing an analgesic role in different inflammatory pain models.

Combined medication analgesia is a widely used analgesic method in clinical practice at present. Through the combined application of analgesic drugs with different action mechanisms, the best curative effect can be obtained by using their additive or synergistic effects [7]. The cholinergic system may interact with the arachidonic acid metabolic pathway. Nevertheless, few studies on the interaction between these two have been reported. In this study, choline and parecoxib sodium were jointly applied to acute and chronic pain models to investigate their analgesic effect and mechanism, thus providing an improved analgesic scheme for the treatment of acute and chronic pain and postoperative pain.

## Materials and methods

### Animals

Adult male Sprague–Dawley (SD) rats (6–8 weeks, 180–220 g) were provided by the Academy of Military Medical Sciences Experimental Animal Center (SCXK-Jun 2012–0004). All experimental protocols were reviewed and approved by the PLA General Hospital Ethics Committee. Animals were acclimated to the testing environment for 2 days. Animals received subcage feeding and were housed (room temperature 20–25 °C, humidity 60–70%) with free access to food and water.

### Materials

Tartaric acid choline (CAS No. 87–67-2, Shanghai Aladdin Biotechnology Co., Ltd., China), parecoxib sodium (Pfizer Ltd., lot: J20080044, USA), pentobarbital sodium (Beijing Chemical Reagent Company, Beijing, China), and 0.9% saline solution (Shijiazhuang Four Drugs Co., Ltd., Hebei) were stored at 4 °C.

### Surgical procedures for chronic constriction injury of the sciatic nerve (CCI)

The rat CCI model was prepared according to the methods described by Bennett et al. [8] Rats were deeply anesthetized with 2% pentobarbital sodium (45 mg/kg, intraperitoneal (i.p.)) and secured on the operating table. Skin in the middle of the left limb was opened after disinfection. The left sciatic nerve was exposed at the mid-thigh level by blunt dissection through the biceps femoris muscle. Four loosely constrictive ligatures (4–0 chrome line, bubble in sterile saline for 30 min) were tied around the nerve near the bifurcation site with a spacing of ~ 1 mm. Nerve ligation was performed slowly, which caused mild fibrillation in the calf muscle but did not block blood circulation. The muscle layer was approximated, and the skin was closed with sutures.

### Drug treatment

Fifty-four rats were randomly divided into 9 groups (*N* = 6/group) (Table 1): sham – rats received sham surgery and saline injection; CCI + saline – rats received CCI surgery and saline treatment; CCI + choline – CCI rats received choline (6, 12, and 24 mg/kg); CCI + parecoxib – CCI rats received parecoxib (3, 6, and 12 mg/kg); and CCI + choline+parecoxib – CCI rats received parecoxib 3 mg/kg and choline 6 mg/kg. Drugs were given by intraperitoneal (i.p.) injection in the morning (9:00–10:00 AM) for 7 days (one injection/day).

**Table 1:** Grouping and treatment

Group	Number	Drug	Intraperitoneal injection
Sham	6	saline	
CCI model	6	saline	
CCI+choline	6	choline	6mg/kg
	6	choline	12mg/kg
	6	choline	24mg/kg
CCI+parecoxib	6	parecoxib	3mg/kg
	6	parecoxib	6mg/kg
	6	parecoxib	12mg/kg
CCI+choline+parecoxib	6	choline+parecoxib	6mg/kg+3mg/kg

### Mechanical and thermal pain threshold tests

Rats were tested on Days 3, 5, 7, 10, and 14 after surgery. The behavior study was conducted between 8:00 ante meridiem (AM) and 5:00 post meridiem (PM). The room temperature was maintained at 20–24 °C. The pain threshold was measured at 30 minutes (min) after each drug injection.

To examine the mechanical pain threshold, rats were placed in an empty cage and remained calm. Mechanical stimulation (von Frey filaments) was applied to the dorsal surface of the left hind paw, which was ipsilateral to the side of nerve injury. Each von Frey filament was applied perpendicularly to the skin to slowly bend the cilia. There was a 30-second (sec) interval between the two tests. If a withdrawal response was evoked, the next stimulus was applied after the rat resettled.

To examine the thermal pain threshold, rats were placed in a plexiglass box, which was placed on a 6-mm-thick glass plate. After 15 minutes of adaptation, the rats were stimulated with a light beam applied to the plantar side of the left hind paw. The time (in sec) between the start of heat stimulation and an evoked paw withdrawal response was measured as the thermal withdrawal latency. The cutoff time was set to 20 sec to prevent tissue damage. The light intensity was set to 40% and kept consistent during the experiment. Each rat was measured 3 times at an interval of 5 minutes, and the data were averaged for analysis.

### Tissue harvest

Another 30 rats were divided into 5 groups (*N* = 6/group): sham (S), CCI + saline (M), CCI + cho-6 mg/kg (P), CCI + pare-3 mg/kg (C), and CCI + cho-6 mg/kg + pare-3 mg/kg (L). The CCI rats received drug treatment for 7 days. Rats were sacrificed after intraperitoneal injection of 2% pentobarbital sodium 45 mg/kg anesthesia on the 8th day, and the L4–6 DRG expanded spinal cord was removed on ice and placed in an EP tube. After labeling, it was quickly placed in a liquid nitrogen tank and frozen at − 80 °C for storage.

#### Western blotting

The DRG tissues from five rats that were randomly selected in each group were used in a western blot study to examine the protein levels of high mobility group protein 1 (HMGB1) and NF-κBp65. The L4–6 DRG was homogenized in RIPA lysis buffer (Servicebio, China) with protease inhibitor and phosphatase inhibitor cocktail (Applygen, Beijing, China). Supernatant protein concentrations were determined after centrifugation at 12,000 rpm for 30 min with a BCA Protein Assay reagent kit (Thermo Pierce, Rockford, IL, USA). Equal amount of the sample (40 μg of protein) was separated on sodium dodecyl sulphate–polyacrylamide gels (SDS–PAGE) and transferred to a polyvinylidenedifluoride (PVDF) membrane. The membranes were incubated with primary antibodies overnight at 4 °C. The primary antibodies were anti-HMGB1 (1:1000; Cell Signaling Technologies), anti-NF-κBp65(1:1000; Cell Signaling Technologies) and anti-β-actin (1:3000; Santa Cruz Biotechnology). The membranes were incubated with anti-mouse or anti-rabbit HRP secondary antibodies (1:3000; Santa Cruz, Biotechnology) for 1 h. The images were digitized from the membrane, and the band intensity was quantified using Gel-Pro Analyzer software, version 3.1 (Media Cybernetics, Bethesda, MD, USA).

### Statistical analysis

All data were analyzed by SPSS 22.0 statistical software. Data are expressed as the mean ± standard error (SE). Intergroup comparisons were conducted by two-way ANOVA (Cho × Pare) followed by Tukey’s post hoc test to determine significant differences between the experimental groups. For the behaviors tested, data were analyzed using a two-way ANOVA (trial days × trial dose) with repeated measures (trial days) followed by a Bonferroni post hoc test to analyze the difference in escape latency between each group. *P* values< 0.05 were considered statistically significant.

## Results

### Dose-dependent pain inhibition produced by choline and parecoxib sodium in CCI rats

#### Mechanical allodynia (von Frey test)

**Fig. 1:**
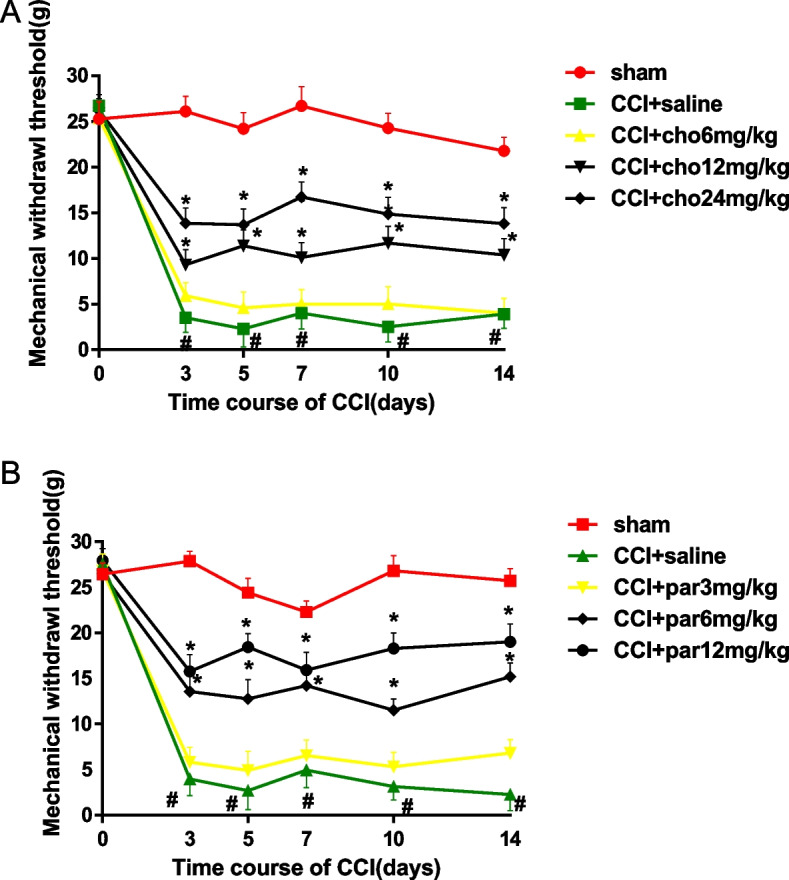
Changes in the paw withdrawal threshold to mechanical stimulation after drug treatment in CCI rats. **A** The mechanical withdrawal threshold of the ipsilateral hind paw was measured at 30 min after intraperitoneal (i.p.) injection of saline or choline (6, 12, and 24 mg/kg) in rats on Days 3, 5, 7, 10 and 14 after chronic constriction injury (CCI). Rats in the sham group only received saline injection. **B** Changes in the mechanical withdrawal threshold after injection of saline or parecoxib sodium (3, 6, and 12 mg/kg, i.p.) in each group. *N* = 6/group. Data are expressed as the mean ± SEM, cho: choline; par: parecoxib sodium. * *P* < 0.05 versus the saline-treated CCI group, # *P* < 0.05 versus Day 0 and the saline-treated sham group

Compared to day 0, saline-treated CCI rats developed mechanical hypersensitivity on the ipsilateral hind paw, as indicated by a significant decrease in the mechanical withdrawal threshold (*P* < 0.001), which occurred at 3 days after injury and persisted until the end of the study. In contrast, saline-treated sham rats did not show mechanical hypersensitivity compared to day 0. During the study, in the drug-treated CCI group, choline and parecoxib produced dose-dependent pain inhibition. Choline significantly inhibited mechanical hypersensitivity in CCI rats at 12 and 24 mg/kg doses compared with that of the CCI group (*P* < 0.05) but not at the 6 mg/kg dose (Fig. 1A). Parecoxib induced significant anti-allodynic effects at 6 and 12 mg/kg doses compared with those of the CCI group (*P* < 0.05) but not at the 3 mg/kg dose (Fig. 1B).

#### Thermal stimulation allodynia

**Fig. 2:**
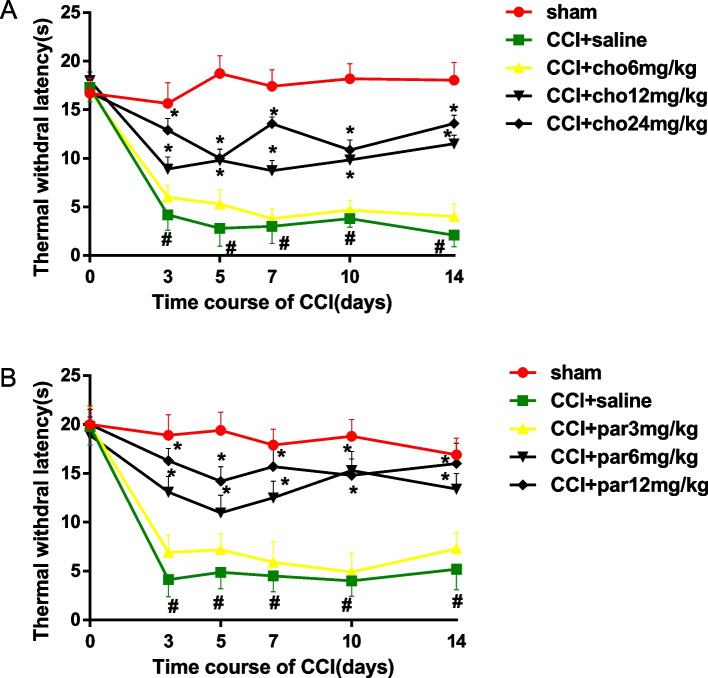
Changes in the thermal withdrawal latency to noxious heat stimulation after drug treatment in CCI rats. **A** The thermal withdrawal latency of the ipsilateral hind paw was measured at 30 min after intraperitoneal (i.p.) injection of saline or choline (6, 12, 24 mg/kg) on Days 3, 5, 7, 10 and 14 after chronic constriction injury (CCI). Rats in the sham group only received saline injection. **B** Changes in thermal withdrawal latency after injection of saline or parecoxib sodium (3, 6, and 12 mg/kg, i.p.) in each group. *N* = 6/group. Data are expressed as the mean ± SEM, cho: choline; par: parecoxib sodium. * *P* < 0.05 versus the saline-treated CCI group, # *P* < 0.05 versus Day 0 and the saline-treated sham group

Compared to day 0, saline-treated CCI rats also developed heat hypersensitivity at 3 days postinjury, which persisted until the end of the study (*P* < 0.001). Saline-treated sham rats did not show heat hypersensitivity compared to day 0. In CCI rats, choline reduced heat hypersensitivity at 12 and 24 mg/kg doses compared with the CCI group (*P* < 0.05) but not at the 6 mg/kg dose (Fig. 2A). Parecoxib reduced heat hypersensitivity at 6 and 12 mg/kg doses compared with the CCI group but not at the 3 mg/kg dose (*P* < 0.05, Fig. 2B).

### Effect of combination therapy

**Fig. 3:**
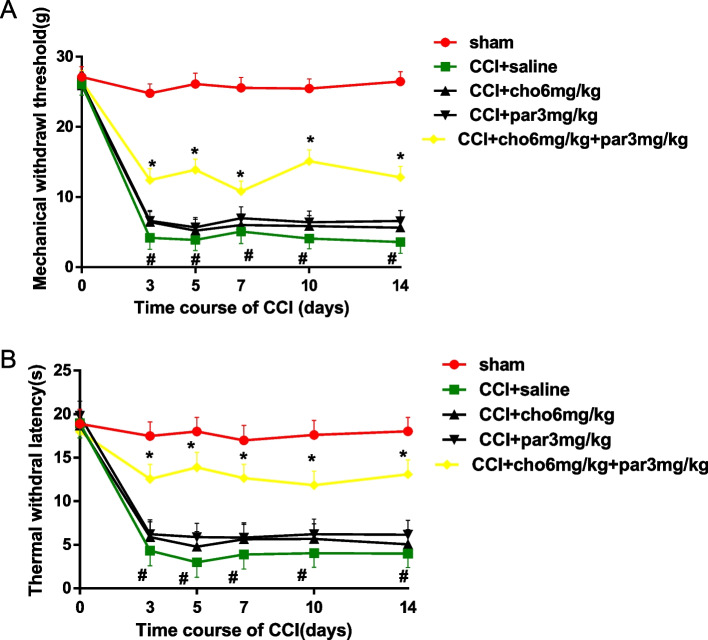
Changes in the mechanical withdrawal threshold and thermal withdrawal latency after individual or combined drug treatment in CCI rats. **A** The paw withdrawal threshold of the ipsilateral hind paw was measured at 30 min after intraperitoneal (i.p.) injection of saline, choline (6 mg/kg), parecoxib sodium (3 mg/kg) or a combination of choline (6 mg/kg) and parecoxib sodium (3 mg/kg) on Days 3, 5, 7, 10 and 14 after chronic constriction injury (CCI). Rats in the sham group only received saline injection. **B** Changes in thermal withdrawal latency in each group after drug treatment. N = 6/group. Data are expressed as the mean ± SEM, cho: choline; par: parecoxib sodium. * *P* < 0.05 versus the saline-treated CCI group, # *P* < 0.05 versus Day 0 and the saline-treated sham group

Compared with the saline-treated CCI rats, choline (6 mg/kg) and parecoxib (3 mg/kg) treatment resulted in a significant inhibition of both mechanical and heat hypersensitivities (*P* < 0.05, Fig. 3A-B).

### Changes in HMGB1 and NF-κBp65 expression in L4–6 DRGs after drug treatment

**Fig. 4:**
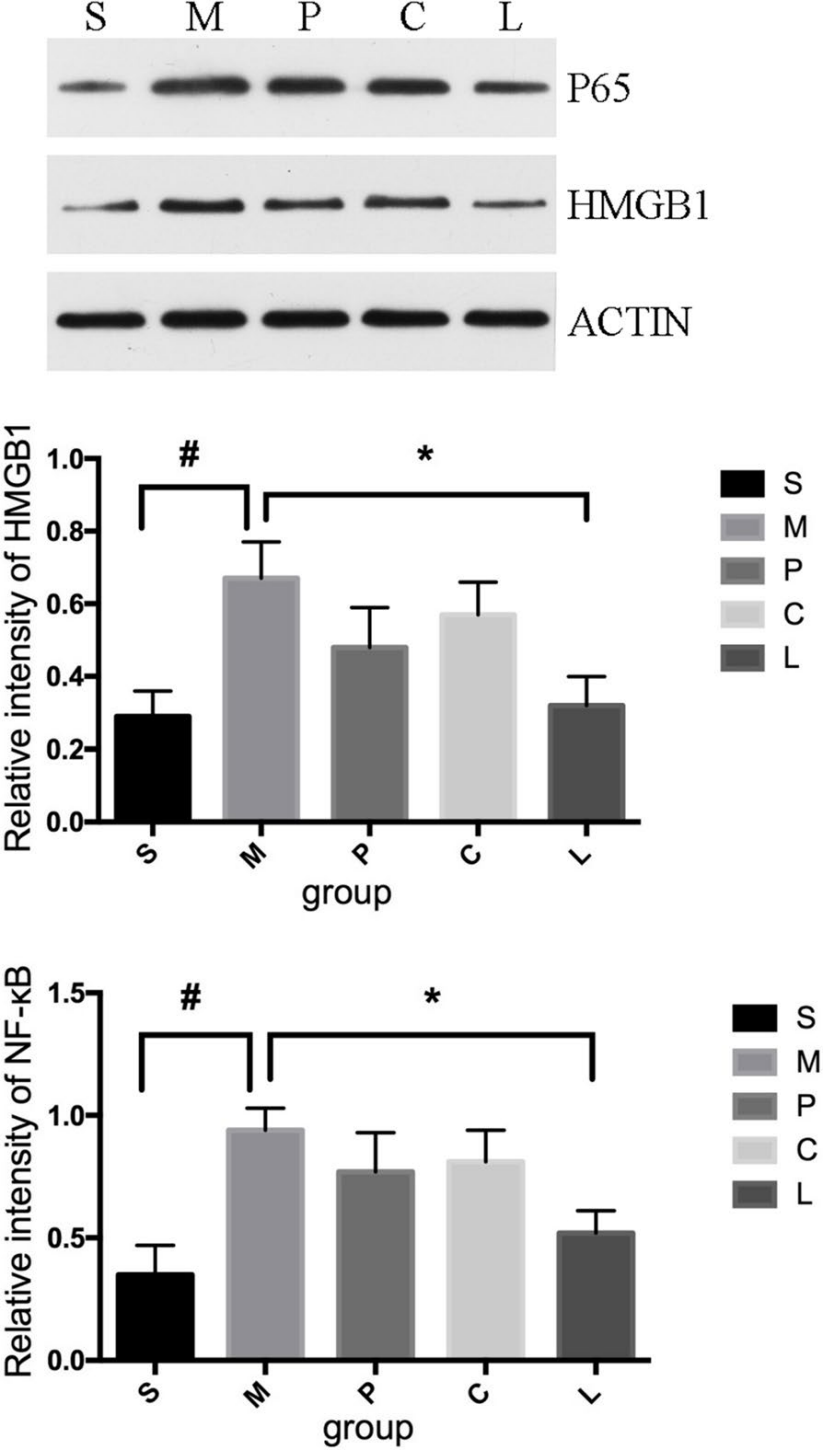
Changes in nuclear factor kappa-B (NF-κB) and high mobility group protein 1 (HMGB1) expression in L4–6 dorsal root ganglia (DRGs) in rats after chronic constriction injury (CCI) of the sciatic nerve and drug treatment. Representative immunoblots and quantification of HMGB1 and NF-κBp65 protein levels in lumbar DRGs (L4–6) of CCI rats at Day 7 after drug treatment. *N* = 5/group. Data are expressed as the mean ± SEM. S: sham, M: CCI + saline, P: CCI + parecoxib-3 mg/kg, C: CCI + choline-6 mg/kg, L: CCI + parecoxib-3 mg/kg + choline-6 mg/kg. * *P* < 0.05, # *P* < 0.05 versus the indicated group

Compared to expression in the sham group, the expression levels of HMGB1 and NF-κBp65 were significantly increased in CCI rats that received saline injection (Fig. 4). Compared with levels in the saline-treated CCI rats, the levels of HMGB1 and NF-κBp65 were significantly decreased in CCI rats that received combined choline (6 mg/kg) and parecoxib (3 mg/kg) treatment for 7 days (*P* < 0.05). However, the levels of HMGB1 and NF-κBp65 were not significantly decreased in CCI rats after receiving single drug treatment with choline (6 mg/kg) or parecoxib (3 mg/kg).

## Discussion

Neuropathic pain is an intractable chronic pain condition. The efficacy of sodium channel inhibitors for treating neuropathic pain has been examined in the past decade [2]. Because current therapies for neuropathic pain are not suitable for some patients, the search for new pain medications and therapies must continue [9].

In this study, we examined the pain inhibitory effects of choline and parecoxib sodium in rats with neuropathic pain. We used the CCI model because it has been reported that this model may simulate some characteristics of neuropathic pain in humans, and CCI of the sciatic nerve induced rapid and significant mechanical and heat hypersensitivities in rats [10]. Similarly, our study showed that CCI rats began to develop pain hypersensitivity at 3 days after injury that persisted through the study period. The affected hind limb (i.e., ipsilateral to the side of CCI) showed eversion and was often lifted to avoid touching the ground. The paw withdrawal threshold to mechanical stimulation and paw withdrawal latency to heat stimulation were significantly decreased compared to those before the injury.

Parecoxib sodium is a highly selective cyclooxygenase-2 (COX-2) inhibitor. It inhibits the production of prostaglandin E2 (PGE2) and nitric oxide and regulates neurotransmission in the spinal cord dorsal horn [11]. Parecoxib sodium has been reported to reduce the accumulation of tumor necrosis factor-α (TNF-α) and neutrophils in inflammatory exudates [12]. Compared to other commonly used nonsteroidal anti-inflammatory drugs (NSAIDs) (e.g., diclofenac and nimesulide), parecoxib sodium induces quicker pain inhibition in humans [13]. Studies have also shown that parecoxib sodium reduced pain and inflammatory responses in formalin and other inflammatory pain models [14]. Our study demonstrated that after 7 days of treatment with 6 mg/kg and 12 mg/kg parecoxib sodium, CCI rats showed significant reductions in mechanical and heat hypersensitivities compared to those of saline-treated CCI rats. However, the 3 mg/kg dose was not effective. Thus, moderate and high doses of parecoxib sodium reduced neuropathic mechanical and heat hypersensitivities, and these findings are consistent with a previous report [15].

As a specific alpha-7 nicotinic acetylcholine receptor (α7nAChR) agonist, choline exerts anti-inflammatory and analgesic effects through activation of the cholinergic anti-inflammatory pathway, [16] which has been shown in several pain models and sepsis models [17]. In an acetic acid torsion model, choline induced dose-dependent analgesic effects [18]. In the current study, choline attenuated mechanical and heat hypersensitivity in CCI rats at 12 mg/kg and 24 mg/kg doses, but 6 mg/kg was not effective. Under chronic neuropathic pain conditions, the decomposition and synthesis of acetylcholine (ACh) are reduced in the hypothalamus, causing a low level of cholinergic activity [19]. Our findings suggest that choline supplementation may help to alleviate chronic pain after nerve injury.

In patients with diabetic neuropathy and postherpetic neuralgia, a previous study showed that morphine in combination with a low dose of gabapentin provided better pain relief than morphine alone [20]. In the current study, cotreatment of CCI rats with a low, subeffective dose of choline (6 mg/kg) and parecoxib sodium (3 mg/kg) induced a significant pain inhibition, indicating that the two drugs may exert an additive or synergistic pain inhibitory effect. Using low doses may limit the dose-limiting adverse effects for each drug.

After peripheral nerve injury, glial cells are activated, and proinflammatory cytokines are released into the spinal cord [21]. HMGB1 is a late inflammatory factor. It can promote the release of proinflammatory factors and increase HMGB1 from inflammatory cells, thus forming a positive feedback loop and aggravating inflammatory responses [22]. NF-κBp65 is an activated nuclear signal transduction protein and mediates the production of inflammatory mediators [23]. Our study showed that the expression levels of HMGB1 and NF-κBp65 were increased in DRGs after CCI, which may increase inflammatory responses and pain.

Parecoxib sodium was shown to reduce the expression of HMGB1 in a cerebral ischemia model, and hence, it plays a neuroprotective role. ^[‘25]^ The choline-activated α7nAChR signaling pathway was suggested to be sensitive enough to control HMGB1 release [24]. In this study, although there was a trend for parecoxib sodium at a 3 mg/kg dose to reduce HMGB1 and NF-κBp65 levels, the difference was not statistically significant. Similarly, choline at a dose of 6 mg/kg did not significantly reduce the upregulation of HMGB1 and NF-κBp65 expression in CCI rats. However, a combination of these drugs significantly reduced the expression of HMGB1 and NF-κBp65, which was consistent with the findings of our behavioral study. HMGB1 can bind and activate Toll-like receptor 4 (TLR4) receptors and induce the production and release of inflammatory factors. NF-κB, as a major nuclear signal transduction protein that mediates the production of a variety of inflammatory mediators, is downstream of the HMGB1/TLR4 pathway [25, 26]. Therefore, these findings suggest that choline and parecoxib sodium may both inhibit the HMGB1/TLR4/NF-κB signaling pathway and may be used together for the treatment of neuropathic pain, which remains a great clinical challenge.

Parecoxib sodium is frequently used in the clinic as an analgesic but has dose-limiting side effects [27]. The cholinergic pathway may also be targeted for pain inhibition by inducing an anti-inflammatory effect [6, 28]. The current findings suggest that combining choline and parecoxib sodium, which have different properties, may improve neuropathic pain inhibition and decrease the side effects of each drug by reducing the required dose. This notion needs to be further tested in future clinical studies.

## Conclusion

1. In a rat model of chronic neuropathic pain (CCI), at a certain dose, choline or parecoxib sodium can alleviate mechanical pain and thermal hyperalgesia caused by CCI.
2. The combination of choline and parecoxib sodium at nonanalgesic doses can effectively relieve neuropathic pain, and its mechanism may be related to the inhibition of the HMGB1/TLR4/NF-κB pathway.

## Supplementary Information

**Additional file 1: Supplementary Figure 1.** Western Blot full-length blots. **Supplementary Figure 2.** S: sham, M: CCI + saline, P: CCI + parecoxib-3 mg/kg, C: CCI + choline-6 mg/kg, L: CCI + parecoxib-3 mg/kg + choline-6 mg/kg.

## Data Availability

The datasets used and/or analyzed during the current study are available from the corresponding author on reasonable request.
