# Supplementary material for: Inhibition effect of choline and parecoxib sodium on chronic constriction nerve injury-induced neuropathic pain in rats
Source: BMC Anesthesiol. 2023 Jan 13;23:22. doi: 10.1186/s12871-022-01913-0 (PMC9837992; doi:10.1186/s12871-022-01913-0)
Supplement: Supplementary file 1 — Additional file 1: Supplementary Figure 1. Western Blot full-length blots. Supplementary Figure 2. S: sham, M: CCI + saline, P: CCI + parecoxib-3 mg/kg, C: CCI + choline-6 mg/kg, L: CCI + parecoxib-3 mg/kg + choline-6 mg/kg. [file 12871_2022_1913_MOESM1_ESM.docx]

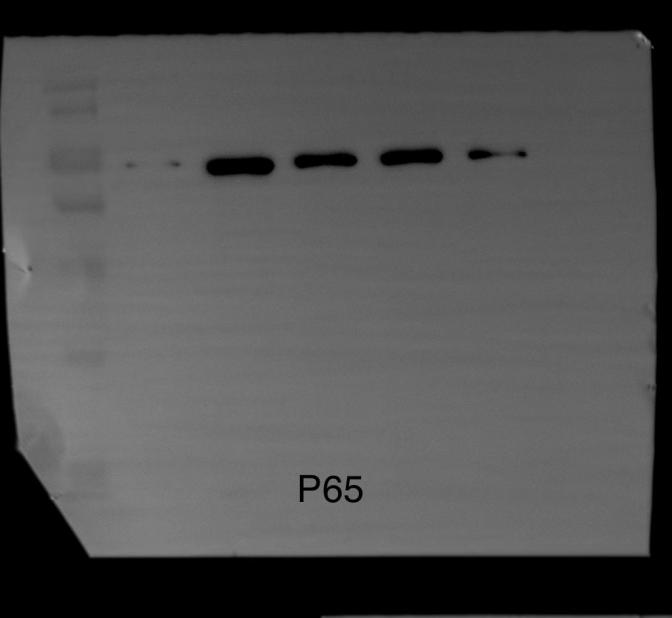

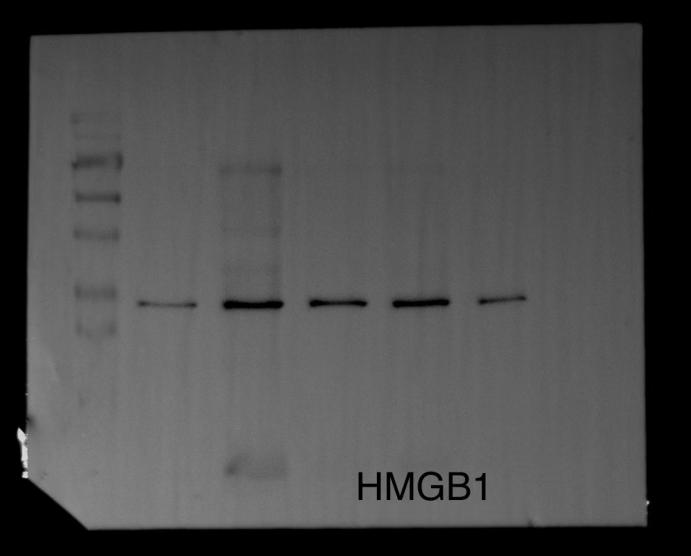


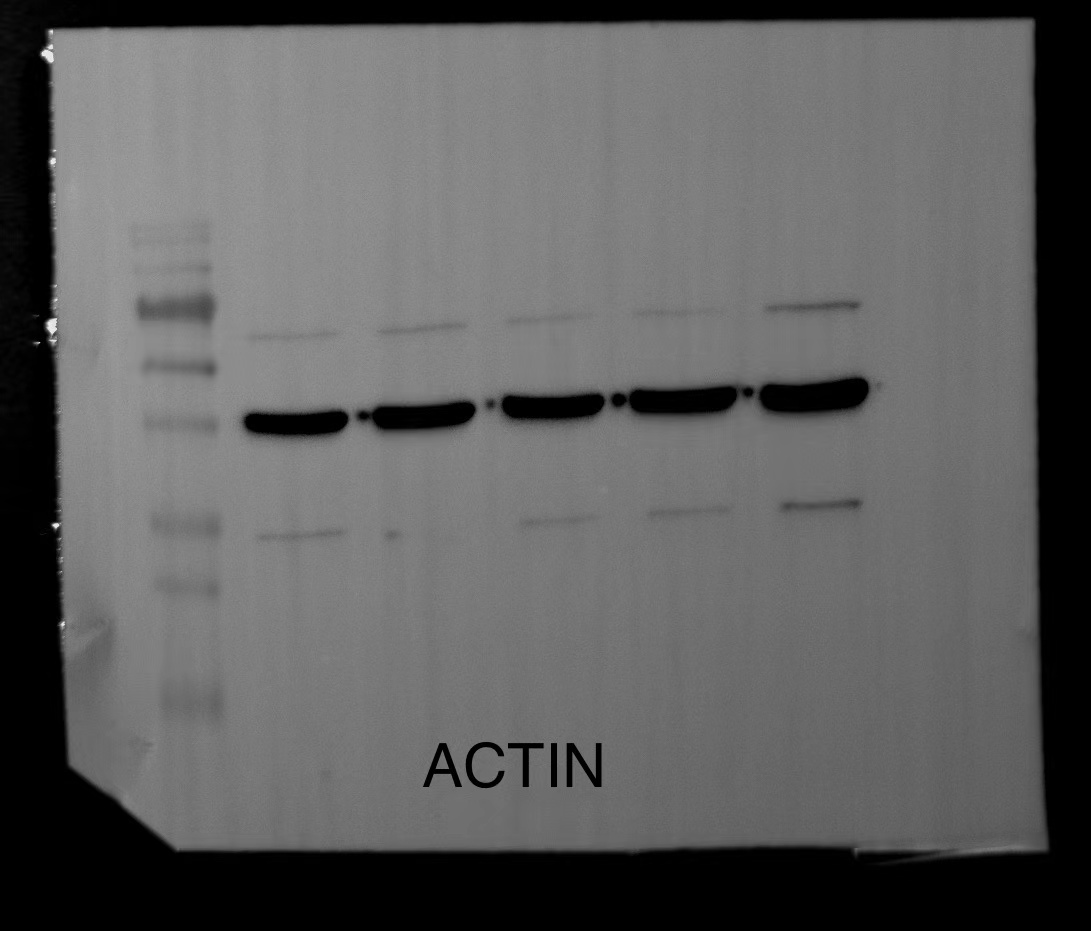


Supplementary figure 1. Western Blot full-length blots.


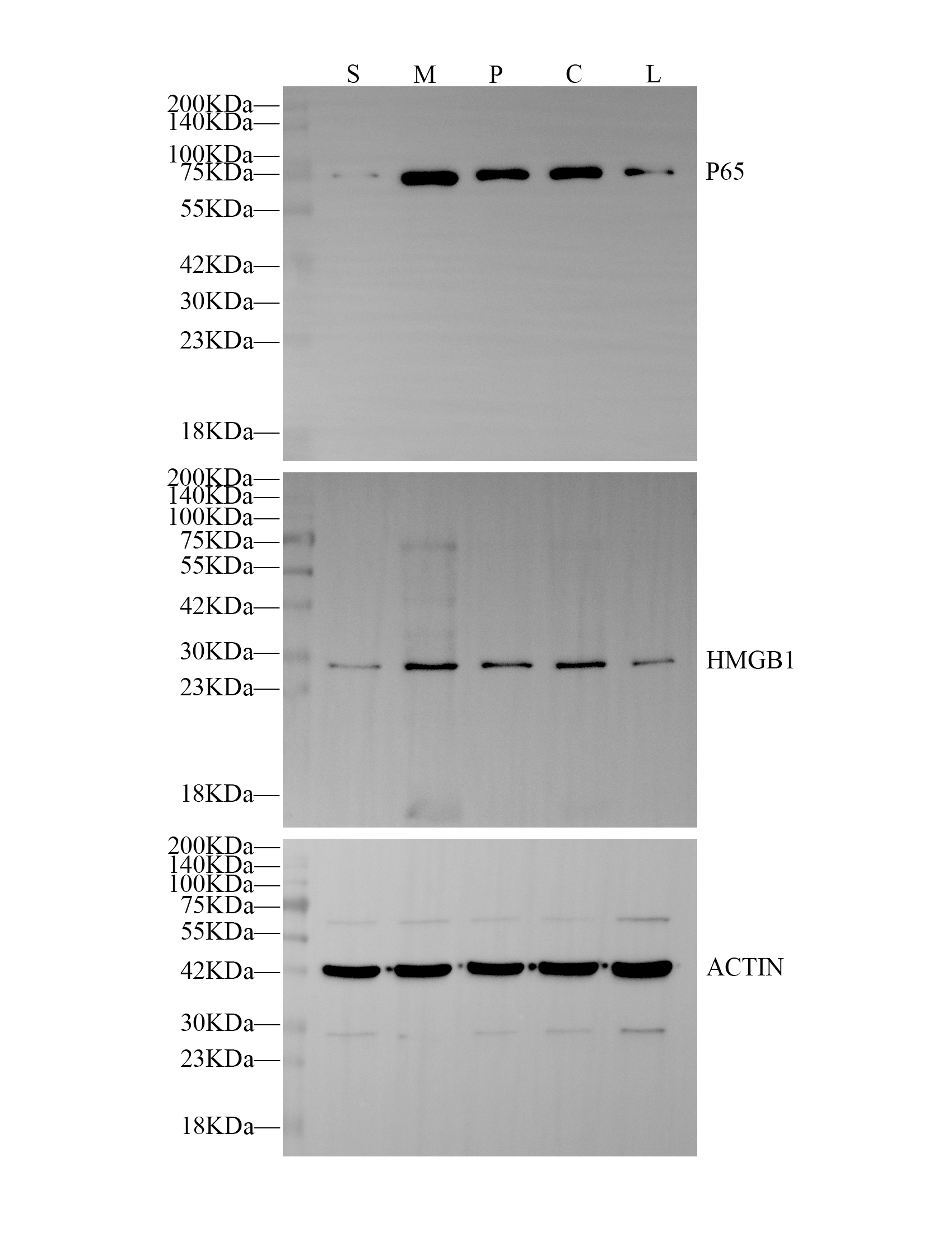


Supplementary figure 2. S: sham, M: CCI+saline, P: CCI+parecoxib-3 mg/kg, C: CCI+choline-6 mg/kg, L: CCI+parecoxib-3 mg/kg+choline-6 mg/kg.
